# Public attitudes in Japan toward the reproductive use of gametes derived from human-induced pluripotent stem cells

**DOI:** 10.2144/fsoa-2021-0065

**Published:** 2021-10-22

**Authors:** Kyoko Akatsuka, Taichi Hatta, Tsutomu Sawai, Misao Fujita

**Affiliations:** 1 Uehiro Research Division for iPS Cell Ethics, Center for iPS Cell Research & Application, Kyoto University, Japan, 53 Kawahara-cho, Shogoin, Sakyo-ku, Kyoto 606-8507, Japan; 2 Shizuoka Graduate University of Public Health, Japan, 4-27-2 Kita Ando, Aoi-ku, Shizuoka 420-0881, Japan; 3 Institute for the Advanced Study of Human Biology (WPI-ASHBi), KUIAS Kyoto University, Japan, Yoshida-Konoe-cho, Sakyo-ku, Kyoto 606-8501, Japan

**Keywords:** assisted reproductive technologies, ethics, human-induced pluripotent stem cells, *in vitro* derived-gametes, *in vitro* gametogenesis, public survey, reproduction

## Abstract

**Purpose:**

To investigate the interests of the Japanese general public in the reproductive use of *in vitro* derived (IVD)-gametes.

**Methods:**

We conducted an online survey and obtained answers from 3096 respondents.

**Results:**

More than half of the respondents agreed with the reproductive use of IVD-gametes by infertile heterosexual married couples but disagreed with the same use by unmarried or homosexual individuals. Nearly 70.0% disagreed with the use for designing a baby.

**Discussion:**

In Japan at present, the use of IVD-gametes that deviate from societal values regarding reproduction and family prescribed by the conventional marriage system is unlikely to be accepted. It is also unlikely to be accepted for non-treatment purposes in reproduction.

Efforts have been made in recent studies to create *in vitro* derived (IVD)-gametes using induced pluripotent stem cells (iPSCs) or embryonic stem cells (ESCs). There are several reports on the successful generation of offspring in mice using IVD-gametes [1,2]. However, the successful generation of human oogonia, which differentiate into ova, was only reported in 2018, and the creation of IVD-gametes has yet to be achieved [3]. The realization of human IVD-gametes would pave the way for those who cannot have children with current assisted reproductive technologies (ARTs), including couples suffering from infertility, to have genetically related children.

However, safety issues have been raised regarding the reproductive use of IVD-gametes. It is uncertain whether these gametes will result in successful pregnancy and childbirth or whether the resulting child and further generations may suffer from some physical harm [4]. Even if there are no physical problems, there is a concern with psychological harm; children born from IVD-gametes may have difficulties in forming a sense of identity [4].

In addition to concerns regarding possible harm imparted to offspring, two major ethical problems have been raised. One considers the determination of eligibility for the reproductive use of IVD-gametes. Technology that enables the creation of gametes from somatic cells is expected to ‘democratize reproduction’ and ‘end infertility’ [4,5]. Logically speaking, *in vitro* gametogenesis (IVG) technology enables anyone to produce genetically related children. IVG technology, therefore, would benefit not just heterosexual couples, but also same-sex couples, post-menopausal women and people who do not want to have a partner but desire to have children. Another ethical issue concerns the scope of the purposes for which to allow the reproductive use of IVD-gametes. Some have pointed out that when combined with preimplantation genetic diagnosis, the technology may be used for eugenic purposes, enabling parents to choose an embryo that has particular physical attributes they wish for the baby [6,7]. In this way, it is theoretically possible to use IVD-gametes for purposes other than so-called ‘infertility treatment’.

Nonetheless, because IVD-gametes creation relies on the use of human embryonic cells and/or fetal cells, it is subject to various technical and ethical constraints, and some estimate that it will take more than 10 years for IVD-gametes to become a reality [8]. Moreover, it is unlikely that IVD-gametes will be accepted as a new option for ART unless all health-related issues are resolved. However, once IVD-gametes are established technologically, we will face the immediate need to determine whether they should be used in reproduction and, in the affirmative case, who is eligible for the treatment and for what purposes [8–10].

Currently, Japan has no established laws specifically regulating the use of ARTs, and medical professionals, both in infertility treatment clinics and general hospitals, instead comply with guidelines issued by the Japan Society of Obstetrics and Gynecology (JSOG). The JSOG currently holds that the use of *in vitro* fertilization (IVF) should be limited to heterosexual married couples who are unable to conceive in other ways. In this context, IVD-gametes will have a significant impact on Japanese and other societies, as they may lead to changes in current values and/or laws and regulations if they are used for reproductive purposes. For this reason, it is important to understand the interests of members of the public who are likely to be affected by future IVG technology in order to prepare for public debates and related policymaking.

Accordingly, this study aimed to understand the general public's interest in the reproductive use of IVD-gametes using a questionnaire survey with three selected topics: who should be allowed to use it, what purposes are acceptable for its use and whether respondents would want to use it personally for reproduction.

## Methods

### Selection of respondents

We prepared a questionnaire and then outsourced the data collection to a survey company. The company has a pool of monitor members that represents the general Japanese population.

Probability sampling with a general sample size requires a sample size of 384 people, provided that the confidence level, margin of error, and response rate are 95, 5 and 0.5%, respectively [11]. To identify the attitudes of the Japanese general public, we classified respondents into six age groups (20–29, 30–39, 40–49, 50–59, 60–69 and 70–79 years old). People under 20 years old or 80 years old or older were excluded from the survey.

Based on the latest available demographic statistics (October 2014) [12], we assigned 384 people to the age 20–29 group, the smallest population group, and estimated the necessary sample size for the other groups, resulting in 481, 543, 463, 546 and 415 people, respectively (2832 in total). The sex distribution of each group was also aligned with that of the corresponding age groups in the demographic data.

While the sample sizes were designed based on probability sampling, the actual sampling process followed a non-probability sampling method using e-mail intervention [13] to form volunteer opt-in panels [11]. To recruit participants for the survey, the survey company sent an e-mail on 17 May 2017, containing the URL of the webpage for informed consent of the survey to all the monitor members who were aged between 20 and 79 years old. The webpage specified the aim of the survey, the estimated time required for answering (10–15 min in total), remuneration for participation (i.e., redeemable points issued by the company), the handling of personal information, and information regarding the executive agency of this survey. The ‘Next’ and ‘Cancel’ buttons were placed at the bottom of the webpage, and when participants clicked the corresponding button they were considered to have given and not given their consent, respectively. Because the number of participants reached the necessary sample size on 18 May 2017, the company disallowed access to the survey through the URL and terminated the recruitment of participants and the collection of answers thereafter. Therefore, complete answers were obtained from 3096 respondents. We do not have a list of respondents because the data we received from the company were anonymized. It must also be noted here that the somewhat larger number of respondents than the sample size that was requested was due to the specifications of the company's recruiting system. The age group and sex distributions were consistent with the reference demographic data. We do not have information on how many individuals were contacted initially, which made it impossible to determine the response rate.

### Ethical considerations

The survey was conducted with the approval of the Joint Medical Ethics Committee of the Institute for Frontier Life and Medical Sciences and the Center for iPS Cell Research and Application, Kyoto University (date of approval: 11 May 2017; approval number 81).

### Content & questionnaire preparation procedures

The distinctive feature of this survey was that the respondents received detailed fact-based explanations about iPSCs and IVG research with supplementary figures and were then asked a wide range of questions (see Supplementary Box 1). This was because, to meet the purpose of this survey, which was to grasp the general public's interests, we considered it important to have the respondents express their attitudes based on accurate information regarding iPSCs and IVG research, topics with which the respondents were unfamiliar. While this paper specifically reports the following seven explanations and questions concerning the reproductive use of IVD-gametes, these are only a subset of a more comprehensive questionnaire that includes other items reported elsewhere [14].

- Fact-based explanation of ‘iPSCs’ and questions regarding the respondents' degree of awareness and interest (explanation 1)
- Fact-based explanation of “research for creating sperm/ova from (human [h])iPSCs,” Supplementary Box 1 & Supplementary Figure 1, and a question regarding the respondents' degree of awareness (explanation 2)
- Explanation of “the production and use of hiPSC-derived sperm/ova,” Supplementary Box 1 & Supplementary Figure 2, and a question regarding the respondents' degree of understanding (explanation 3)
- A question asking who the respondents think should be eligible for the reproductive use of hiPSC-derived gametes (degree of acceptance by users) (question 4)
- A question asking for what purposes the respondents think the reproductive use of hiPSC-derived gametes should be used (degree of acceptance by purposes) (question 5)
- A question asking whether the respondents may be interested in using hiPSC-derived gametes to produce their own children (preference) (question 6)
- Questions regarding the respondents' demographic characteristics (question 7)

Explanations 1 to 3 were designed to measure, as a baseline, the respondents' previous knowledge about and interests in iPSCs and IVG research to identify the degree of their understanding of the provided fact-based explanations, and to investigate the degree to which this knowledge and understanding influenced the participants' attitudes toward those studies.

Regarding the reproductive use of IVD-gametes, we devised questions 4 and 5, which concern the degrees of acceptance by type of user and by type of purpose, respectively, based on preceding arguments in bioethics [4,9,15]. This allowed for more detail of the extent to which the respondents would accept such technologies.

Question 4 presented seven stereotypical types of potential users who could potentially access IVG technology in the future and asked the respondents about their attitudes toward each type with the question, “To what degree do you agree that the people described below use hiPSC-derived sperm/ova to produce children?” The seven types were as follows: heterosexual married couples without the need for infertility treatments; heterosexual married couples undergoing infertility treatments; heterosexual married couples who cannot expect successful infertility treatments; heterosexual married couples who cannot have children due to menopause; heterosexual unmarried couples without the need for infertility treatments; a man or a woman without or preferring not to have a partner; and a homosexual couple (gay or lesbian). For the user type “a heterosexual married couple who cannot expect successful infertility treatments,” we added the following explanatory note: “people for whom conventional infertility treatments are ineffective due to their inability to produce sperm or ova caused by, for example, past cancer treatments they received in their childhood.”

Question 5 presented four possible purposes for the use of IVD-gametes and asked the respondents about their attitudes toward each with the question, “To what degree do you agree with the use of hiPSC-derived sperm/ova to produce children for each of the following purposes?” The purposes were: “to avoid passing hereditary disorders to the child,” “to use this technology to enable conception” for treatment purposes, “to produce children with specific appearances or abilities” for a non-treatment purpose, and “to produce children resistant to diseases” for a prophylactic purpose with treatment and non-treatment aspects. For the purpose “to avoid passing hereditary disorders to the child,” we added the following explanatory note: “Huntington's disease and some types of hemophilia are caused by genetic abnormalities, and when the parents possess the gene responsible, it has a certain probability of being inherited by their children. This purpose refers to such genetic diseases caused by abnormalities in specific genes.”

The questions regarding the degrees of acceptance by users and by purposes were provided with five answer options, including four options on a 4-point Likert scale (“1. I do not agree at all” to “4. I strongly agree”) and an additional “5. I don't know.”

Question 6 concerned the respondents' preferences and asked “Would you consider using hiPSC-derived sperm/ova to produce your child?” to identify their attitudes toward the reproductive use of IVD-gametes for themselves instead of their use by other people. This question was also provided with five answer options, including four options on a 4-point Likert scale (“1. I never want to use it” to “4. I strongly want to use it”) and an additional “5. I do not know.”

Regarding demographic characteristics, we used the registration data obtained from the survey company concerning age, sex, marital status and personal income. For other characteristics, including educational background, presence or absence of children, religious affiliations, and experience of infertility treatment, we asked question 7.

In preparing the questionnaire, we consulted science communicators who had received life science education regarding the questionnaire's readability. In addition, we asked scientists involved in IVG research to offer advice from a scientific perspective regarding the explanations for the creation and use of IVD-gametes, the design of the supplementary figures, and the appropriateness of the questions.

### Data analysis

In this study, we conducted descriptive statistics to determine the general public's attitudes toward iPSCs and IVG research. Since the general public may be unfamiliar with the topics of the survey, it was assumed that a proportion of respondents would select ‘I don't know’ for questions about the degree of acceptance by users, by purposes, and personal preference (questions 4–6). Given this possibility, we calculated the Cohen's kappa coefficient (k factor) as the concordance rate that one respondent would choose ‘I don't know’ for two distinct questions after converting the survey responses to questions 4 and 5 into two subsets: one for ‘I don't know’ and the other for all other options. Higher concordance rates may be construed if the respondents had an ambiguous attitude toward this highly novel topic of IVD-gamete use in general rather than not having clear opinions about specific questions.

For the statistical analyses reported herein, we used IBM SPSS Regression 24.0 (IBM Corp., NY, USA) and Microsoft Office Professional Plus 2016 Excel (Microsoft Corp., WA, USA).

## Results

### Demographic characteristics of the respondents

We obtained complete answers from 3096 respondents. The respondents' demographic characteristics and their degree of awareness, interest, and understanding regarding iPSCs and IVG research are shown in Table 1 and published elsewhere [14].

**Table 1.** Demographic characteristics of the respondents (N = 3096).

	n	%
Sex		
Male	1533	49.5
Female	1563	50.5
Age (years)		
20–29	419	13.5
30–39	525	17.0
40–49	593	19.2
50–59	507	16.4
60–69	597	19.3
70–79	455	14.7
Marital status		
Married	2066	66.7
Unmarried	1030	33.3
Presence or absence of children		
Yes	1871	60.4
No	1225	39.6
Experience of infertility treatment		
No	2772	89.5
Yes	261	8.4
I do not want to answer	63	2.0
Educational background		
Elementary school	4	0.1
Junior high school	75	2.4
High school	1041	33.6
Technical college	351	11.3
Two-year college	327	10.6
Four-year college	1159	37.4
Postgraduate studies (master's degree)	105	3.4
Postgraduate studies (doctorate)	23	0.7
Other	11	0.4
Personal income (yen/year)		
Less than two million	1175	38
Two to four million	698	22.5
Four to six million	410	13.2
Six to eight million	185	6.0
Eight to ten million	66	2.1
Ten to twelve million	33	1.1
Twelve to fifty million	7	0.2
Fifty to twenty million	6	0.2
Over twenty million	7	0.2
I do not know	195	6.3
I do not want to answer	314	10.1
Religious affiliation		
No, I do not have a religious affiliation	2646	85.5
Yes, I have a religious affiliation	313	10.1
I do not want to answer	137	4.4
How much did you know about iPSCs?		
I knew enough about them to be able to provide an explanation to a certain extent	607	19.6
I had only heard a bit about them	2267	73.2
I was not aware of them	222	7.2
Are you interested in iPSCs?		
Yes, I am interested	2220	71.7
No, I am not interested	876	28.3
How much did you know about the “research for creating sperm/ova from hiPSCs”?		
I knew enough about it to be able to provide an explanation to a certain extent	212	6.8
I had only heard a bit about it	1322	42.7
I was not aware of it	1562	50.5
How much did you understand the explanation on “the creation and use of hiPSC-derived sperm/ova”?		
I fully understood it	128	4.1
I mostly understood it	2101	67.9
I could not understand it very well	743	24.0
I could not understand it at all	124	4.0

hiPSC: Human-induced pluripotent stem cell; iPSC: Induced pluripotent stem cell.

### Degree of acceptance by user

**Figure 1.**
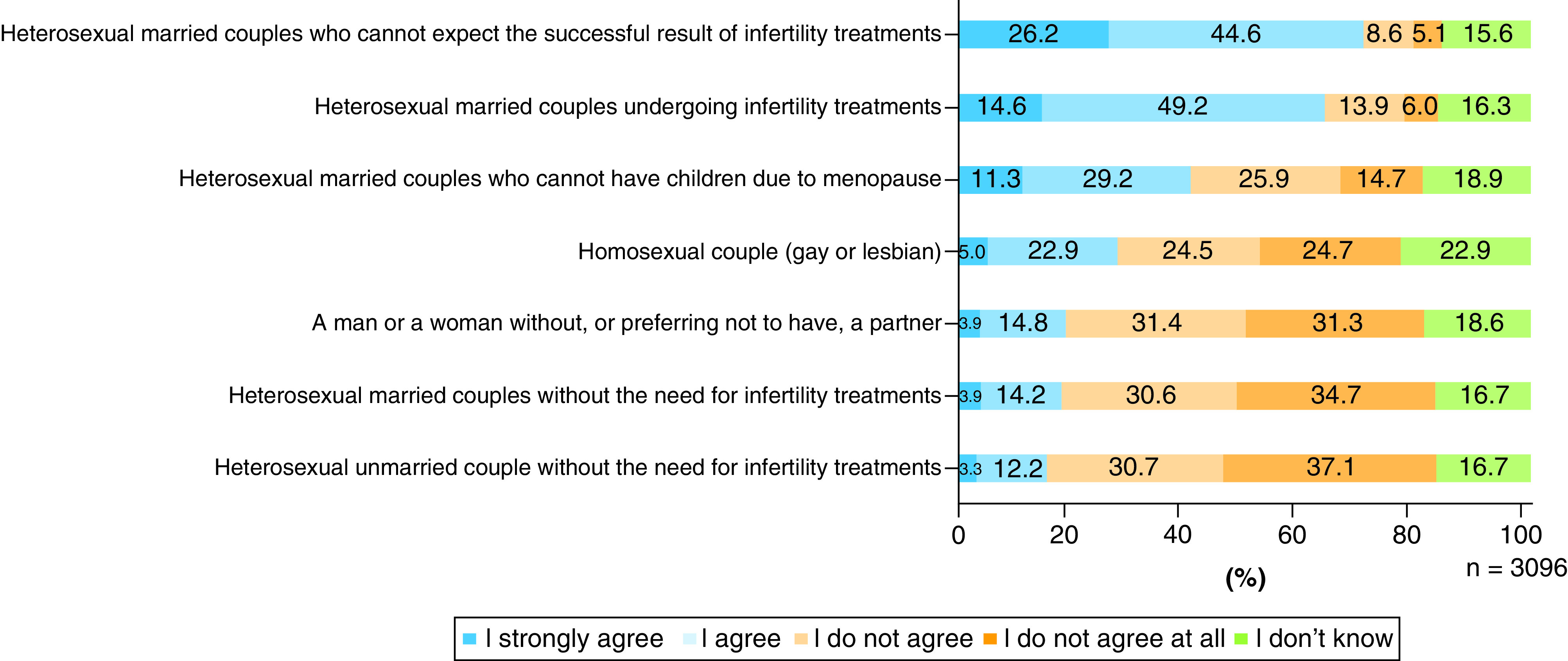
Attitude of acceptance by user type concerning the reproductive use of IVD-gametes.

For “a heterosexual married couple who cannot expect successful infertility treatments” and “a heterosexual married couple undergoing infertility treatments,” 70.8 and 63.8% of the respondents agreed on the reproductive use of IVD-gametes, respectively. Regarding ‘a homosexual couple’, 49.2% disagreed with the use of the technology, while those who agreed were only 27.9%. As for “a man or a woman without or preferring not to have a partner,” less than 20% of the respondents answered in the affirmative, and 60% were in opposition (Figure 1).

About 20% of the respondents selected ‘I don't know’ for each user type. The concordance rate of this answer between user types revealed a *k* factor between 0.556 and 0.851 (Table 2). Thus, the concordance rate between user types is widespread, covering ‘moderate’ (0.41 < *k* <0.60) to ‘almost perfect’ (0.81 < *k*) [16]. This suggests that a person who answered ‘I don't know’ about one of the IVD-gamete user types provided in this question was mildly likely to choose ‘I don't know’ as an answer for other user types.

**Table 2.** Concordance rates of ‘I don't know’ answers for acceptance by user type (Cohen's kappa coefficient).

		Q10_1_DK	Q10_2_DK	Q10_3_DK	Q10_4_DK	Q10_5_DK	Q10_6_DK
Q10_1_DK	Heterosexual married couple not needing infertility treatment						
Q10_2_DK	Heterosexual married couple undergoing infertility treatment	0.668					
Q10_3_DK	Heterosexual married couple unable to expect successful results of infertility treatment	0.662	0.851				
Q10_4_DK	Heterosexual married couple unable to have children due to menopause	0.627	0.683	0.689			
Q10_5_DK	Heterosexual unmarried couple not needing infertility treatment	0.793	0.656	0.662	0.686		
Q10_6_DK	Person without, or preferring not to have, a partner	0.689	0.591	0.605	0.665	0.791	
Q10_7_DK	Same-sex couple (gay/lesbian)	0.556	0.554	0.555	0.636	0.611	0.715

### Degree of acceptance by purpose

**Figure 2.**
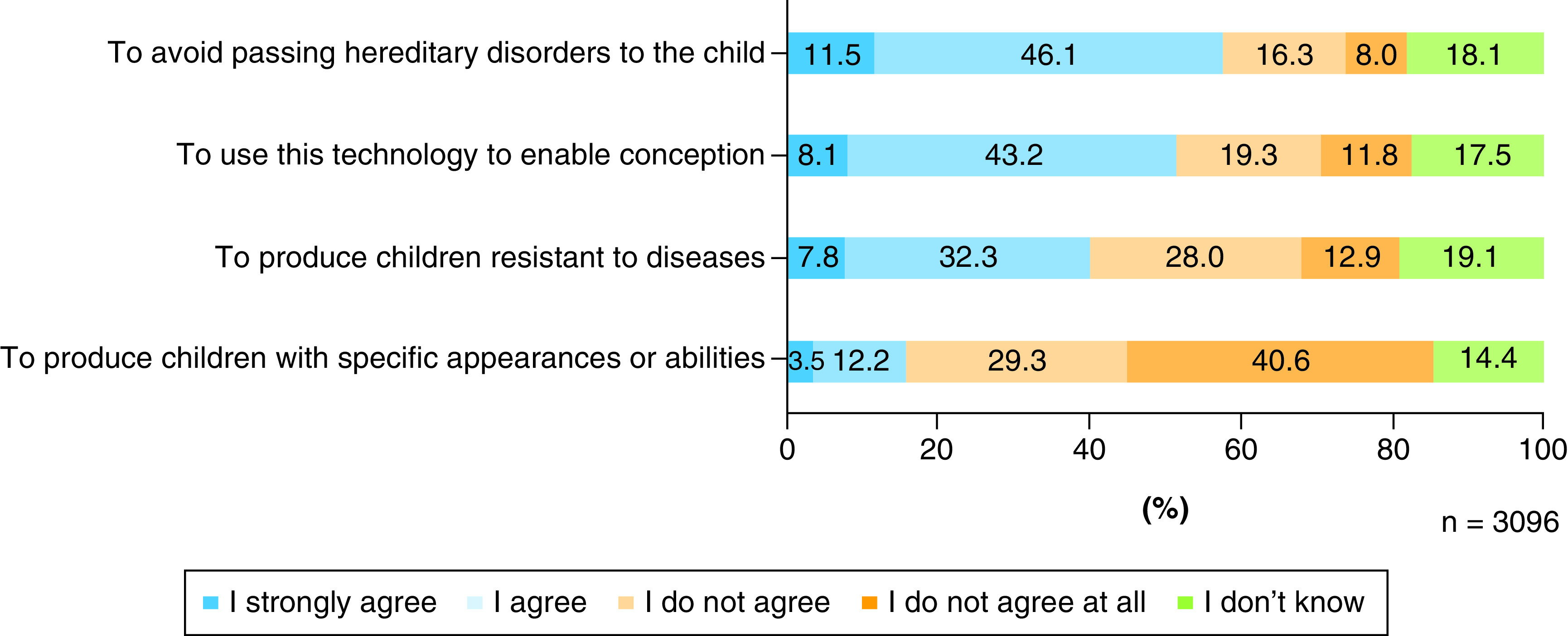
Attitude of acceptance by purpose type concerning the reproductive use of IVD-gametes.

More than half of the respondents agreed “to avoid passing hereditary disorders to the child” and “to use this technology to enable conception” (Figure 2), but approximately 70% disagreed with the use of the technology “to produce children with specific appearances or abilities.”

Again, for each purpose, about 20% of the respondents chose ‘I don't know’. The concordance rate of this answer between the purpose types revealed a *k* factor between 0.636 and 0.734 (Table 3). Given the substantial concordance (.61 < k <.80) between any purpose type [16], there is a tendency that a person who answered “I don't know” about one of the purposes of IVD-gamete use provided in this question chose the answer ‘I don't know’ for other purpose types.

**Table 3.** Concordance rates of ‘I don't know’ answers for acceptance by purpose type (Cohen's kappa coefficient).

		Q11_1_DK	Q11_2_DK	Q11_3_DK
Q11_1_DK	To use this technology to enable conception			
Q11_2_DK	To avoid passing hereditary disorders to the child	0.674		
Q11_3_DK	To produce children resistant to diseases	0.672	0.734	
Q11_4_DK	To produce children with specific appearance or abilities	0.642	0.636	0.681

### Respondents' personal preferences

**Figure 3.**
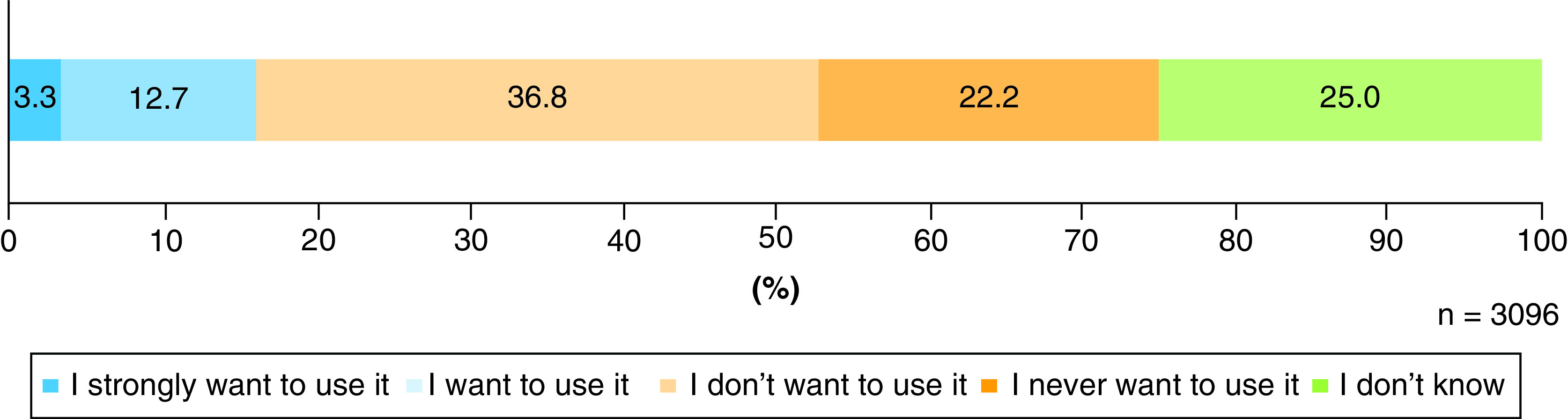
Personal preferences concerning the reproductive use of IVD-gametes.

Regarding the question of whether the respondents would be interested in using IVD-gametes to have their own children, 16.0% answered “I want to use it”, 59.0% answered “I don't want to use it” and 25.0% answered “I do not know” (Figure 3).

## Discussion

### Respondents' attitudes toward the use of IVD-gametes for reproductive purposes correspond with values regarding family & reproduction in the Japanese marriage system

The fact that many respondents agreed with the use of IVD-gametes for reproductive purposes by married couples who were either undergoing infertility treatments or expecting unsuccessful results from such treatments suggests that those respondents regarded the reproductive use of IVD-gametes by these people is in line with infertility treatments such as IVF and artificial insemination (AI). In contrast, many respondents opposed the use of this technology by homosexual couples and people without or preferring not to have a partner, indicating the possibility that these respondents considered the use of IVD-gametes for reproductive purposes by such users as out of the scope of infertility treatment. These attitudes correspond to the present circumstances in Japan, which approves infertility treatments only for heterosexual married couples of reproductive ages [17].

One research group reported similar survey results in some respects. Hendriks *et al.* conducted a study between 2016 and 2017 in The Netherlands, asking obstetricians (n = 82) and the general public (n = 772) among which of eight potential IVD-gamete user groups they would approve the reproductive use of IVD-gametes. The identified acceptance levels (obstetricians, general people) were as follows: 1. Young heterosexual couples in which the women are infertile (99%, 94%); 2. Young heterosexual couples in which the men are infertile (97%, 94%); 3. Infertile single women considering the use of donated sperm (91%, 69%); 4. Female same-sex couples (61%, 68%); 5. Male same-sex couples considering surrogacy (50%, 62%); 6. Heterosexual couples suffering from infertility of an unknown cause (43%, 83%); 7. Fertile women wishing to have a child who is genetically related only to her (6%, 27%); and 8. Heterosexual couples in which the woman is post-menopausal (26%, 17%) [18]. Compared with our research results, the respondents of the study by Hendriks *et al.* overall presented remarkably high levels of acceptance regarding the reproductive use of IVD-gametes. The tendencies of the highest level of acceptance for infertile couples and lower levels of acceptance for fertile single individuals are in agreement with the results of our study, while a difference is found in terms of their high levels of acceptance of the use of IVD-gametes by male or female same-sex couples.

These similarities and differences may be attributed to differences in Japanese and Dutch values concerning regulations and views on reproductive medicine and infertility, views on same-sex couples or single individuals having children, and the perception of the relationship between marriage and reproduction. First, Japan does not legally recognize same-sex marriage [19]; thus, homosexual couples cannot legally be parents, although some local authorities have now introduced same-sex partnership programs. Second, there are no established laws concerning the use of ART in Japan. In practice, guidelines operated by the JSOG are used to regulate reproductive medicine in Japan. The JSOG guidelines do not allow people other than infertile couples to access ART [20,21]. Third, it has been reported that the Japanese public is less approving of providing people other than infertile couples with access to donated sperm/ova [22], suggesting a lack of social acceptance for the pursuit of childrearing by singles and same-sex couples. Fourth, according to 2015 data, children born outside of marriage only accounted for 2% of all newborns in Japan [23]. This indicates that for most people in Japan, childbirth is experienced after marriage, while very few people become unmarried parents.

In contrast, reproductive liberty is guaranteed in The Netherlands, where legal frameworks have been developed with consideration for various potential cases of conceiving and giving birth to a child using ART [24]. The country also strives for the welfare of children born through technology. For example, it is mandatory to ensure the preservation and disclosure of information about gamete donors [25–27]. Compared with Japan, there is a more accepting attitude in The Netherlands toward a diversity of family structures that go beyond marital relationships and heterosexual parents. Furthermore, according to statistics, the birth rate of children outside of marriage in The Netherlands has been increasing by 10% over the last 10 years, and more than 50% of children in the country were born through conception outside of marriage as of 2018, which indicates that the Dutch association of marital relationships with childrearing is not as strong as it is with the Japanese [28,29]. Thus, people's attitudes in terms of who should be eligible for the reproductive use of IVD-gametes is considered to strongly reflect the differences in values, social systems, and marital and reproductive status between the two countries.

Regarding the results concerning the degree of acceptance by users, an interesting finding was observed in terms of attitudes toward post-menopausal couples. The present study found that 40% of respondents approved of IVD-gamete use for reproduction by a heterosexual married couple who cannot have children due to menopause, but another 40% were against, signifying divided opinions. This may be because those respondents who accepted the use of IVD-gametes by couples undergoing or unable to expect successful results of infertility treatments either regarded the infertility treatment as something that could be extended to post-menopausal women or it would be out of the scope of general infertility treatments. While it is unclear the age of post-menopausal married couples the respondents assumed, it is unlikely they believed IVD-gamete use would be for infertility treatment if they assumed the couples were in their late-40s or older. In this sense, while the present study cannot make firm conclusions, there is the possibility that some respondents found it less acceptable for older post-menopausal women to use this technology. It is also possible that the list of potential users prompted the respondents to consider whether post-menopausal couples should be allowed to use IVD-gametes to have children compared with other types of users.

### The Japanese public show a higher degree of acceptance of the reproductive use of IVD-gametes for treatment purposes

In the survey, we presented cases of conceiving a child and avoiding certain hereditary disorders caused by specific genetic abnormalities, such as Huntington's disease and some types of hemophilia, as the purpose of using IVD-gametes, which more than half of the respondents accepted. This result suggests that the reproductive use of IVD-gametes for treatment purposes may be acceptable to many people.

Meanwhile, the reproductive use of IVD-gametes for non-treatment purposes resulted in lower degrees of acceptance. Regarding the purpose of producing children with specific physical attributes or abilities, 69.9% of respondents expressed disagreement, and more than half of these 69.9% answered “strongly disagree,” expressing their categorical disapproval. As explained above, a bioethicist argues that IVD-gametes could be used in reproductive settings for eugenic purposes beyond general medical treatment purposes [7]. Considering the results of the present study, many people at present find it difficult to accept the reproductive use of IVD-gametes for non-treatment purposes.

As for the use “to produce children resistant to diseases,” 40.1% of respondents expressed their acceptance, while 40.9% disagreed with the idea, indicating divided opinions. The percentage of 40.1% is lower than the acceptance level for use as a treatment (57.6%), but higher than the case for the non-treatment purpose “to produce children with specific appearances or abilities (15.7%).” Based on these findings, it is difficult to determine at present whether the use of IVD-gametes in reproduction to prevent disease is socially acceptable.

Moreover, understanding treatment, non-treatment, and preventive purposes (a combination of treatment and non-treatment) may depend on the times, culture, and/or geographical region. Some argue that the treatment/non-treatment definition of an intervention also depends on the theories that form the basis of health and illness [30]. The results of the present study suggest that the reproductive use of IVD-gametes for preventive purposes is controversial; therefore, even when the reproductive use of IVD-gametes for treatment purposes is legally acceptable, deliberations are necessary to determine what kinds of purposes are socially regarded as a medical intervention and how the use for preventive purposes should be permitted.

### People who have experienced infertility treatment are more likely to accept the reproductive use of IVD-gametes

In the personal preference question, less than 20% of respondents expressed interest in using IVD-gametes personally to have a child. Given this, we assumed that a certain number of respondents were of the opinion that the reproductive use of IVD-gametes was acceptable if used by infertile couples wanting to have children but were not inclined to use it themselves. Based on this assumption, we compared the answers between a subgroup of respondents aged 49 years or younger who had experienced infertility treatments (n = 139), since we expected them to have a higher preference for the reproductive use of IVD-gametes, and all other respondents (n = 2957). We identified a marked difference between these subgroups in their preference regarding the reproductive use of IVD-gametes for themselves: the first subgroup was more likely to express a personal preference to use this technology (*x*^2^ = 14.61; df = 2; p = .001; Supplementary Table 1).

Similarly, we compared these two subgroups regarding the degree of acceptance by purpose and found that the first subgroup was more likely to agree with the idea of “using the technology to enable conception” (*x*^2^ = 34.77; df = 2; p = 0.000). Interestingly, this subgroup (n = 139) was also more likely to accept the use of the technology “to produce children with specific appearances or abilities” (*x*^2^ = 14.61; df = 2; p = 0.001). There were no differences between the subgroups in terms of their responses to the purposes “to avoid passing hereditary disorders to the child” and “to produce children resistant to diseases” (Supplementary Table 2).

These analyses were conducted in addition to the original study design based on the interest that arose from the analytical results of the descriptive statistics. Because these questions were not intended to explain the hypotheses we formulated for the study, we did not include this content in the Results section and instead placed it in the Discussions section. However, the above results indicate that the data collected in this study provided insights into personal involvement in reproduction. In order to consider whether in the future IVD-gametes will be accepted as an ART currently practiced in Japan, such as AI and IVF, further research is required to investigate how personal involvement in reproduction may influence opinions on the reproductive use of IVD-gametes.

### Limitations & significance of our survey

Because the survey for this study was conducted by monitor members of an online survey company, the respondents were necessarily limited to those who use the internet and excluded those who do not. In this sense, the responses may have been biased if being an internet user is a significant variable influencing attitudes toward IVG technology. While this was a limitation of this study, statistics from the Ministry of Internal Affairs and Communications suggest that internet usage is widespread in Japan; as of 2019, more than 70% of the population aged 70 to 79 used the internet frequently [31]. Based on this, we considered email to be an acceptable means of reaching the general population. In fact, we considered email as the most appropriate means to recruit participants, given that those who responded to the survey request were subscribed members of an online survey service company and were assumed to be accustomed to answering online surveys. Moreover, the survey was designed with a sampling method that minimized sex and age biases in respondents' answers by sampling in alignment with the demographic distribution of sex and age in the Japanese population, and the data were gathered from more than 3000 members of the general public. Thus, the findings of the study should maintain a certain degree of generalizability of the Japanese general public at the time the survey was implemented.

Although more than 3 years have passed since the survey was conducted, it is acceptable to assume that the public attitude toward IVG technology has not changed significantly when considering that during the period from the survey implementation until today no major development has been observed in Japan in terms of IVG technology and policy debates concerning it. Therefore, the passage of time after the survey is unlikely to affect the results or the discussions presented in this paper.

In this study, we investigated the attitude of the Japanese general public toward the reproductive use of IVD gametes. Based on discussions on the degrees of acceptance by user type and by purpose type, our results suggest that it will be difficult to find social acceptability for IVD-gametes in modern Japan for uses that contradict current values regarding reproduction and family that presuppose the marriage system or for non-treatment purposes. Thus, at present, the results suggest it will be difficult for this technology to gain acceptance for use by individuals who do not need to undergo infertility treatments in order to produce genetically related children or for cases in which, even for heterosexual married couples in need of infertility treatments, the technology is used to produce a child with particular attributes or abilities beyond infertility treatment purposes.

It is worth noting that the respondents may have found the questions difficult to answer due to a lack of familiarity with the subject matter even though they were provided a basic explanation in advance. Some respondents, therefore, may have been influenced by their values and opinions about conventional reproductive techniques, such as IVF, when answering the survey. Future studies should explore how respondents' general attitudes toward prevalent reproductive techniques affect their attitude toward the use of IVD-gametes.

The study also falls short in terms of analyzing the correlation between respondents' sociodemographic attributes and their attitudes. These analyses might produce interesting insights about sociodemographic characteristics. However, to conduct such an analysis, we would need to handle a large volume of data and include more objective and explanatory variables. Because our main objective in this study was to describe which user and purpose types are acceptable for the use of IVD-gametes rather than who approves the use of the technique, we felt that the additional analysis would deviate from the objectives and purposes of this study. Therefore, we believe that these analyses should be included in future studies.

## Conclusion

Overall, the attitudes of the general public elucidated by this survey will serve as a source for discussions once IVG technology is truly established, not only with regards to whether IVD-gametes should be accepted for reproductive purposes, but also more specifically, who should be allowed to use the technology and for what kind of purposes. In addition, longitudinal research on the degrees of acceptance by user type and purpose type would be useful for understanding the changes in the ethical and social acceptability of IVD-gamete use for reproductive purposes and for holding discussions reflecting the current situation.

**Summary points**

- The reproductive use of IVD-gametes will have a significant impact on society because of its potential to change existing social values, laws, and regulations. Public debates involving a diversity of stakeholders, including the general public, are thus required regarding their use.
- While it is important to understand the interests of the general public in the use of IVD-gametes for reproductive purposes, few attitude surveys have been conducted to investigate this matter, and no such survey has ever been conducted in Japan.
- In this study, an online survey was conducted in May 2017, with the general public as the target population, and 3096 respondents participated.
- In response to questions regarding who should be accepted as users of IVD-gametes for reproductive purposes, more than 60% of respondents expressed their acceptance of its use by heterosexual married couples undergoing infertility treatments or those unable to expect successful results from these treatments, while less than 30% of respondents found the use by same-sex couples and single individuals acceptable.
- In response to the question about what purposes are acceptable for using IVD-gametes for reproduction, more than half of respondents answered that they would accept it for producing genetically related children and to avoid passing hereditary disorders to the child, but less than 20% of respondents found it acceptable to use the technology to produce children with certain appearances or abilities desired by the parents.
- In response to the question concerning whether the respondents wanted to use IVD-gametes to have their own children, about 10% of them answered positively, but 60% had stated they did not want to use the technology personally.
- The results of the survey suggest that the Japanese general public is unlikely to accept the use of IVD-gametes in ways that deviate from the shared values of family or reproduction that presuppose the current marriage system or from conventional treatment purposes.
- Further research of a longitudinal manner is needed to support public debate on the reproductive use of IVD-gametes.

## References

[1] Hayashi K, Ohta H, Kurimoto K Reconstitution of the mouse germ cell specification pathway in culture by pluripotent stem cells. Cell 146(6), 519–532 (2011). 21820164 10.1016/j.cell.2011.06.052

[2] Hayashi K, Ogushi S, Kurimoto K Offspring from oocytes derived from *in vitro* primordial germ cell-like cells in mice. Science 338(6109), 971–975 (2012). 23042295 10.1126/science.1226889

[3] Yamashiro C, Sasaki K, Yabuta Y Generation of human oogonia from induced pluripotent stem cells *in vitro*. Science 362(6412), 356–360 (2018). 30237246 10.1126/science.aat1674

[4] Smadjor A, Cutas D. Artificial gametes. Nuffield Council on Bioethics: Background Paper (2015). https://www.nuffieldbioethics.org/wp-content/uploads/Background-paper-2016-Artificial-gametes.pdf

[5] Testa G, Harris J. Ethical aspects of ES cell-derived gametes. Science 305(5691), 1719 (2004). 15375251 10.1126/science.1103083

[6] Bourne H, Douglas T, Savulescu J. Procreative beneficence and *in vitro* gametogenesis. Monash Bioeth. Rev. 30(2), 29–48 (2012). 23409535 10.1007/BF03351338 PMC3590899

[7] Sparrow R. *In vitro* eugenics. J. Med. Ethics 40(11), 725–731 (2014). 23557913 10.1136/medethics-2012-101200

[8] Ishii T, Saitou M. Promoting *In Vitro* gametogenesis research with a social understanding. Trends Mol. Med. 23(11), 985–988 (2017). 29032005 10.1016/j.molmed.2017.09.006

[9] Bredenoord AL, Hyun I. Ethics of stem cell-derived gametes made in a dish: fertility for everyone? EMBO Mol. Med. 9(4), 396–398 (2017). 28279974 10.15252/emmm.201607291 PMC5376744

[10] Cohen IG, Daley GQ, Adashi EY. Disruptive reproductive technologies. Sci. Transl. Med. 9(372), 1–3 (2017). 10.1126/scitranslmed.aag2959 28077678

[11] Sue VM, Ritter LA. Conducting the Surveys. In: Conducting Online Surveys. Sue VM, Ritter LA (Eds). SAGE Publications, Inc., CA, USA, 88–98 (2007).

[12] The Bureau of Statistics of the Ministry of Internal Affairs and Communications. The Japan Population Estimates 2015. (2015). https://www.stat.go.jp/data/jinsui/2014np/index.html

[13] Sue VM, Ritter LA. Sampling. In: Conducting Online Surveys. Sue VM, Ritter LA (Eds). SAGE Publications, Inc., CA, USA, 25–37 (2007).

[14] Sawai T, Hatta T, Akatsuka K, Fujita M. Public attitudes in Japan toward the creation and use of gametes derived from human-induced pluripotent stem cells. Future Sci. OA (2021) (Epub ahead of print). 10.2144/fsoa-2021-0066 PMC8610011 34840812

[15] Smajdor A, Cutas D. Will artificial gametes end infertility?. Health Care Anal. 23(2), 134–147 (2015b). 24293033 10.1007/s10728-013-0268-x

[16] Landis J, Koch G. The measurement of observer agreement for categorical data. Biometrics 33(1), 159–174 (1977). 843571

[17] Japan Society of Obstetrics and Gynecology (JSOG). Infertility (2018). http://www.jsog.or.jp/modules/diseases/index.php?content_id=15

[18] Hendriks S, Dancet EAF, Vliegenthart R, Repping S. The acceptability of stem cell-based fertility treatments for different indications. Mol. Hum. Reprod. 23(12), 855–863 (2017). 28460040 10.1093/molehr/gax027

[19] The Constitution of Japan (Article 24). https://japan.kantei.go.jp/constitution_and_government_of_japan/constitution_e.html

[20] Japan Society of Obstetrics and Gynecology (JSOG). Views on *in vitro* fertilization and embryo transfer. (2014). http://www.jsog.or.jp/modules/statement/index.php?content_id=20

[21] Japan Society of Obstetrics and Gynecology (JSOG). Views on artificial insemination using donated sperm. (2015). http://www.jsog.or.jp/modules/statement/index.php?content_id=24

[22] Yamamoto N, Hirata T, Izumi G A survey of public attitudes towards third-party reproduction in Japan in 2014. PLoS ONE 13(10), e0198499 (2018). 30379816 10.1371/journal.pone.0198499 PMC6209135

[23] National Institute of Population and Social Security Research. Marriage process and fertility of married couples attitudes toward marriage and family among Japanese singles: highlights of survey results on married couples/singles. (2017). http://www.ipss.go.jp/ps-doukou/e/doukou15/Nfs15R_points_eng.pdf

[24] Calhaz-Jorge C, De Geyter Ch, Kupka MS Survey on ART and IUI: legislation, regulation, funding and registries in European countries: The European IVF-monitoring Consortium (EIM) for the European Society of Human Reproduction and Embryology (ESHRE). Hum. Reprod. Open. 2020(1), (2020). 10.1093/hropen/hoz044 PMC7002185 32042927

[25] Bolt S, Postema D, van der Heij A, Maas B M AJ. Anonymous Dutch sperm donors releasing their identity. Hum. Fertil. 17, 1–7 (2019). 10.1080/14647273.2018.1564156 30652500

[26] Janssens PMW, Simons AHM, van Kooij RJ A new Dutch Law regulating provision of identifying information of donors to offspring: background, content and impact. Hum. Reprod. 21(4), 852–856 (2005). 16339167 10.1093/humrep/dei407

[27] Visser M, Mochtar MH, de Melker AA Psychosocial counselling of identifiable sperm donors. Hum. Reprod. 31(5), 1066–1074 (2016). 26975325 10.1093/humrep/dew037

[28] Eurostat. Share of live births outside marriage. (2018). https://ec.europa.eu/eurostat/tgm/table.do?tab=table&init=1&language=en&pcode=tps00018&plugin=1

[29] OECD. Share of births outside of marriage. http://www.oecd.org/els/family/SF_2_4_Share_births_outside_marriage.pdf

[30] Notoni L, Gyngell C, Savulescu J. Drawing the line on *in vitro* gametogenesis. Bioethics 34(1), 123–134 (2019). 31617217 10.1111/bioe.12679 PMC6973109

[31] The Ministry of Internal Affairs and Communications. Results of FY2019 Communication Usage Trend Survey. https://www.soumu.go.jp/johotsusintokei/statistics/data/200529_1.pdf

